# Relationship between quality of professional capacity building for kindergarten teachers and children’s language development: the mediating role of kindergarten language education activities quality

**DOI:** 10.3389/fpsyg.2023.1219330

**Published:** 2023-08-04

**Authors:** Qiong Wu, Guoxia Wang, Chang Li

**Affiliations:** ^1^Faculty of Education, Northeast Normal University, Changchun, Jilin, China; ^2^School of Psychology, Northeast Normal University, Changchun, Jilin, China

**Keywords:** children’s language development level, quality of professional capacity building for kindergarten teachers, kindergarten language education activities quality, teacher training, preschool education reform

## Abstract

Strengthening the professional capacity building for kindergarten teachers is crucial for improving the quality of kindergarten education and promoting children’s development. In order to explore the impact of the quality of professional capacity building for kindergarten teachers and kindergarten language education activities quality on children’s language development, a study was conducted. A total of 1,584 children from 90 kindergartens in 5 provinces in China were randomly selected as research participants. Utilizing an independent sample *t*-test and correlation analysis (with SPSS 26.0 as the tool), the study analyzed the disparities in the professional capacity building for kindergarten teachers of different types, kindergarten language education activities quality, and the children’s language developmental levels, as well as the interrelationships among these factors. A multilevel structural equation model (using MPLUS 8.3 as the tool) was employed to analyze the mediating role that the kindergarten language education activities quality plays between the quality of professional capacity building for kindergarten teachers and children’s language development levels. The results indicate that children in public kindergartens demonstrate significantly higher language development levels than those in private kindergartens. A positive correlation exists between the quality of professional capacity building for kindergarten teachers, kindergarten language education activities quality and children’s language development levels. Furthermore, kindergarten language education activities quality shows partial mediating effects on the relationship between them. These findings highlight the need to enhance the quality of education in private kindergartens, reduce the disparities in language development levels between private and public kindergartens, and reinforce the quality of professional capacity building for kindergarten teachers to provide effective support for children’s language development. Ultimately, the study suggests concentrating on the organization and implementation of kindergarten language education activities as a means of evaluating the quality of professional capacity building for kindergarten teachers.

## 1. Introduction

Strengthening teacher professional development is a key step towards implementing the connotation construction and achieving high-quality development in preschool education in China. Since 2010, China has issued a series of policy documents that provide specific opinions and requirements on how to build the kindergarten teacher workforce. In 2012, the Ministry of Education issued the “Professional Standards for Kindergarten Teachers (Trial Implementation),” which positioned “ability as the priority” as one of the basic principles that kindergarten teachers must adhere to (Ministry of Education of the People’s Republic of China, 2012). In 2018, the Central Committee of the Communist Party of China and the State Council issued the “Opinions on Deepening the Reform of Teacher Development in the New Era,” which emphasized the need to effectively enhance the professional childcare and education skills of kindergarten teachers (The State Council of the CPC Central Committee, 2018). In 2022, the Ministry of Education issued a notice on the “Guidelines for Evaluating the Quality of Preschool Care and Education,” which included key indicators for evaluating the teacher workforce such as professional development, staffing, professional ethics, and incentive mechanisms, with the aim of strengthening the ethical work of kindergarten teachers and emphasizing their professional development (Ministry of Education of the People’s Republic of China, 2022). It is evident that China is currently emphasizing the professional development of kindergarten teachers, focusing on improving their professionalization level, and promoting the in-depth development of preschool education reform.

The developmental status of children is an important indicator for measuring the quality of professional capacity building of kindergarten teachers and the success of preschool education reform (Hong and Pang, 2006; Li et al., 2021). Against the backdrop of China’s emphasis on the quality of professional capacity building for kindergarten teachers, it is a highly concerning issue for policy makers and researchers to explore whether the quality of professional capacity building for kindergarten teachers can promote children’s development and to what extent. The main approaches to professional capacity building for kindergarten teachers include teacher training, teaching and research activities, incentives and evaluation, and autonomous development (Xiao and Hu, 2007; Wang, 2011; Xu and Yun, 2013; Sun and Zhang, 2019). Currently, there have been some empirical studies abroad exploring the relationship between teacher training and child development. A meta-analysis study on the impact of teacher professional development program quality on child development suggests that teacher training has a significant effect on both teacher professional growth and child development (Egert et al., 2018). Teacher training and support in building intimate teacher-child relationships can maximize children’s readiness for learning by promoting social/emotional, behavioral, cognitive, and physical health development (Smith and Glass, 2019). When innovative methods such as after-school consultation, online face-to-face guidance or supervision are used to train teachers, it can effectively improve the quality of the course implementation process and promote the early math skills of preschool children (Domitrovich et al., 2009; Kinzie et al., 2014). Compared with teachers who did not receive professional development programs, those who received project training made significant overall progress in teacher-student communication (Wasik et al., 2006) and were better able to plan and think of appropriate professional measures to support children’s language development (Michel et al., 2014). Children who interacted with teachers participating in professional development programs were more efficient in language and used more complex language than those who interacted with teachers using traditional methods (Piasta et al., 2012), and their knowledge of grammar and letters was also expanded (Cabell et al., 2011). Increasing expenditure on kindergarten teacher training is an effective measure to improve the quality of the education process and has a positive impact on children’s development and learning (Hu et al., 2017). However, some studies have suggested that there is no relationship between teacher training and child development (Lin and Magnuson, 2018). This may be due to the fact that teacher training has not provided teachers with the necessary knowledge and skills to promote child development (Hyson et al., 2009). Another possible reason is that in low-income social environments, due to limited educational resources, teachers may not be able to apply the knowledge and skills learned from training in the classroom (Wang et al., 2020).

Teaching and research activities refer to practical and reflective research activities in which kindergarten teachers analyze and solve specific problems faced in education and teaching in a planned, process-oriented, and methodical manner under the organization of research leaders, with the purpose of promoting teacher professional development. Previous studies have analyzed the value of teacher research activities and found that they are of great value in promoting the comprehensive development of young children (Torquati et al., 2007), but there is a lack of evidence-based educational research in this area. Researchers mainly focus on the implementation, existing problems, and implementation strategies of policy requirements for teacher motivation and evaluation, as well as teacher autonomous development, but there are very few studies on their effectiveness. As a result, although various methods have been adopted to promote the professional development of kindergarten teachers in practice, the actual effectiveness, including their impact on child development, is unknown. Moreover, it is unclear which aspects of the existing content and pathways of teacher professional development need to be improved to effectively enhance teacher professional development.

Therefore, it is imperative to conduct in-depth research on the relationship between the quality of professional capacity building for kindergarten teachers and children’s development. Current research on the relationship between teacher training and child development is inconclusive, and it is necessary to conduct a thorough analysis of the relationship between the quality of professional capacity building for kindergarten teachers in other forms and children’s development in kindergartens. Child development refers to the comprehensive development of children in areas such as physical, cognitive, emotional, social adaptation, and language (UNICEF, 2017). Language is an important indicator for assessing children’s developmental level. In different countries and regions, language development is considered an important area or key dimension in guidelines for children’s learning and development and in assessments of child development (OECD, 2020).

Teacher professional capacity building aims at promoting the professional development for kindergarten teachers that can improve the quality of kindergarten education activities. Kindergarten language education activities refers to various forms of teaching process carried out in kindergartens with children as the subject and language as the object (Zhang, 2010). Studies have shown that kindergarten language education activities can systematically and efficiently help children acquire the core experience of language learning and development (Wang et al., 2021). According to global quality measures, young children in high-quality educational activities tend to score higher on receptive vocabulary and better language skills (Abreu-Lima et al., 2013). Kindergarten education activities focusing on literacy can improve children’s early literacy skills (Farver et al., 2009; Goodrich et al., 2017).

This study aims to explore the relationship between the quality of professional capacity building for kindergarten teachers and children’s language development, and to analyze the mediating role of kindergarten language education activities quality. The findings of this study will provide scientific evidence for improving the quality of professional capacity building for kindergarten teachers and promoting children’s language development.

This study proposes the following research hypotheses: Firstly, there are significant differences in language development levels among children in different types of kindergartens (public and private). Secondly, the quality of professional capacity building for kindergarten teachers significantly affects children’s language development levels. Thirdly, kindergarten language education activities quality plays a mediating role between the quality of professional capacity building for kindergarten teachers and children’s language development levels.

## 2. Research methods

### 2.1. Research participants

This study employed a stratified sampling method to select 100 public and private kindergartens in urban, suburban, town, and rural areas in five provinces (autonomous regions) of China: Guangxi, Jilin, Jiangsu, Shaanxi, and Zhejiang. One junior class (3–4 years old children), middle class (4–5 years old children) and senior class (5–6 years old children) were randomly selected from each kindergarten, and 6 children (half male and half female) were randomly sampled from each class, resulting in a total of 1,800 children. Based on the research questions to be explored, 10 kindergartens with missing data on quality of professional capacity building for kindergarten teachers were excluded, a total of 90 kindergartens with an effective rate of 90% were selected as the study sample. The sample size for child language development assessment was 1,584 from 90 kindergartens (The evaluation results of 36 young children were incorrect, so they were deleted), with an effective rate of 98%. Among them, 911 children were from public kindergartens, and 673 were from private kindergartens (see Table 1 for details).

**Table 1:** Description of sampled kindergartens and children.

Province	Kindergarten ownership		Number of kindergartens	Sample classes			Number of children
	Public	Private		Junior class of kindergarten	Middle class of kindergarten	Senior class of kindergarten	
Guangxi	11	9	20	115	117	118	350
Jilin	6	6	12	62	64	69	195
Jiangsu	13	7	20	120	118	117	355
Shaanxi	10	8	18	108	108	108	324
Zhejiang	12	8	20	118	119	123	360
Total	52	38	90	523	526	535	1,584

### 2.2. Evaluation instruments

#### 2.2.1. Evaluation tool for quality of professional capacity building for kindergarten teachers

The study employed the evaluation tool for assessing the quality of professional capacity building for kindergarten teachers, based on the “The Path Towards Excellence—Chinese Kindergarten Education Quality Rating Standards (short for PTE-CKEQRS in the following) (Chen et al., 2021). The overall and internal consistency of the PTE-CKEQRS as well as the consistency of each domain, reached a high level. Most items demonstrated good discriminant validity and construct validity. The PTE-CKEQRS consist of five quality domains: *Management Leadership*, *Environmental Support*, *Curriculum Promotion*, *Teacher Qualifications Guarantee* and *Home-Kindergarten-Community Cooperation*. Each domain contains several items, sub-items, and finely graded indicators of different levels. For example, *Teacher Qualifications Guarantee* comprises four items: teacher staffing, salary benefits, teacher ethics, and professional development. The professional development item includes four sub-items: teacher training, teaching and research activities, incentives and evaluations, and autonomous development, each with several finely graded evaluation indicators of different levels. In the quality evaluation process, evaluators first assess each fine indicator, and the evaluation result is either yes or no. Then, they score the sub-item using the Likert seven point scoring method, where 1 point represents not applicable, 3 points represent qualified, 5 points represent good, and 7 points represent excellent. The sub-item score follows the principle of step-by-step inference from low to high, assigning a corresponding level of score (between 1 and 7) based on the evaluation results and scoring rules of the fine indicators. The item score is the average score of multiple sub-items, ranging from 1 to 7 (with two decimal places). A higher score indicates higher quality of professional capacity building for kindergarten teachers. The internal consistency reliability of the tool is good, with a Cronbach’s alpha coefficient of 0.849.

#### 2.2.2. Evaluation tool for kindergarten language education activities quality

This study evaluated kindergarten language education activities quality using the language domain of the “Curriculum Promotion” quality area in the PTE-CKEQRS (Chen et al., 2021). The language domain includes four sub-items: listening, speaking, reading, and writing. The scoring of kindergarten language education activities quality is the same as that of the quality of professional capacity building for kindergarten teachers. The internal consistency reliability of this tool is good, with Cronbach’s alpha coefficients for the language domain ranging from 0.754.

#### 2.2.3. Evaluation tool for children’s language development level

The tool used to assess children’s language development level is the Peabody Picture Vocabulary Test-revised edition type A (PPVT) (Lu and Liu, 2005). The PPVT adopts a 0–1 scoring system, with a score of 1 for correct answers and 0 for incorrect answers. Studies have shown that this assessment tool has good psychometric properties (Zhang et al., 2021), with internal consistency reliability coefficients of 0.91.

### 2.3. Evaluation process

Prior to the formal evaluation, researchers underwent rigorous and standardized testing training, which included evaluation methods, interpretation of evaluation indicators, and on-site testing. For the evaluation of quality of professional capacity building for kindergarten teachers and kindergarten language education activity quality, two evaluators worked independently in each group. After the evaluation was completed, they discussed and made the final evaluation results based on the PTE-CKEQRS. For the assessment of children’s language development level, child development evaluators conducted one-on-one assessments on sampled children in the kindergarten. The assessment guidelines and procedures were strictly followed according to each measurement scale’s instructions. The assessment usually took 10–20 min depending on the child’s age and response time.

### 2.4. Data analysis

This study used SPSS 26.0 for independent samples *t*-test and correlation analysis. As the independent variable (quality of professional capacity building for kindergarten teachers) and the mediating variable (kindergarten language education activities quality) in this study belong to the kindergarten level (L2), while the dependent variable (children’s language development level) belongs to the individual level (L1), a multilevel data analysis method was adopted. The analysis of mediating effects used the coefficient product method, which examines whether *a* × *b* is significant. If *a* × *b* is significant (significant level of 0.05) and its confidence interval does not include 0, it indicates a significant multilevel mediating effect (Zhao et al., 2010). Mplus 8.3 was used as the analysis software.

## 3. Results and analysis

### 3.1. Means and standard deviations of all study variables

Kindergartens can be classified into public kindergartens and private kindergartens based on their ownership. An independent samples *t*-test was conducted to examine the differences in children’s language development level, quality of professional capacity building for kindergarten teachers, kindergarten language education activities quality between different types of kindergartens (see Table 2 for results). The results showed significant differences in children’s language development level, quality of professional capacity building for kindergarten teachers, and kindergarten language education activities quality between different types of kindergartens. Children attending public kindergartens had significantly higher language development levels than those attending private kindergartens (*t* = 3.56, *p* <.01), the quality of professional capacity building for kindergarten teachers and kindergarten language education activity quality in public kindergarten had significantly higher than those in private kindergartens (*t* = 17.79, *p* < .001; *t* = 9.714, *p* < .001).

**Table 2:** Means and standard deviations of all study variables.

Evaluation Indicator	Public		Private		*t*
	Mean	SD	Mean	SD	
Children’s language development level	52.83	23.54	48.50	23.96	3.56**
Quality of professional capacity building for kindergarten teachers	4.58	1.58	3.57	1.10	17.79***
Kindergarten language education activities quality	3.61	0.75	3.27	0.56	9.714***

**p < 0.01; ***p < 0.001.

### 3.2. Correlation analysis of all study variables

The raw scores of children’s language development levels in each age group were transformed into standard scores. Then, Pearson correlation was used to examine the correlation between quality of professional capacity building for kindergarten teachers in the four dimensions and children’s language development level (results shown in Table 3). The statistical results showed that the quality of teacher training, teaching and research activities, incentives and evaluation, autonomous development were all significantly positively correlated with children’s language development level and kindergarten language education activities quality.

**Table 3:** Correlation analysis of all study variables.

Variables	Correlation							Mean ± SD
	1	2	3	4	5	6	7	
1. Quality of professional capacity building for kindergarten teachers	1							4.147 ± 1.223
2. Teacher training	0.786**	1						3.022 ± 2.034
3. Teaching and research activities	0.766**	0.414**	1					5.39 ± 1.547
4. Incentives and evaluation	0.673**	0.358**	0.384**	1				4.025 ± 1.212
5. Autonomous development	0.814**	0.466**	0.562**	0.479**	1			4.152 ± 1.587
6. Children’s language development level	0.222**	0.157**	0.171**	0.153**	0.201**	1		0.025 ± 0.983
7. Kindergarten language education activities quality	0.396**	0.299**	0.310**	0.297**	0.309**	0.232**	1	3.465 ± 0.696

**p < 0.01.

### 3.3. Mediating effect test

As the quality of professional capacity building for kindergarten teachers and kindergarten language education activities quality belong to the level of kindergarten hierarchy (L2), while children’s language development level belongs to the level of individual development (L1), a multilevel structural equation modeling approach is adopted for the subsequent mediation analysis. Firstly, the null model of children’s language development level is tested to examine whether the data is suitable for multilevel analysis. The result shows that the within-group correlation coefficient (ICC) of children’s language development level is 0.194, which is greater than 0.06, indicating the necessity of conducting multilevel analysis on the explanatory mechanism of the antecedents of children’s language development level. A 2-2-1 model is constructed, which includes quality of professional capacity building for kindergarten teachers (L2) – kindergarten language education activities quality (L2) – children’s language development level (L1).

**Figure 1:**
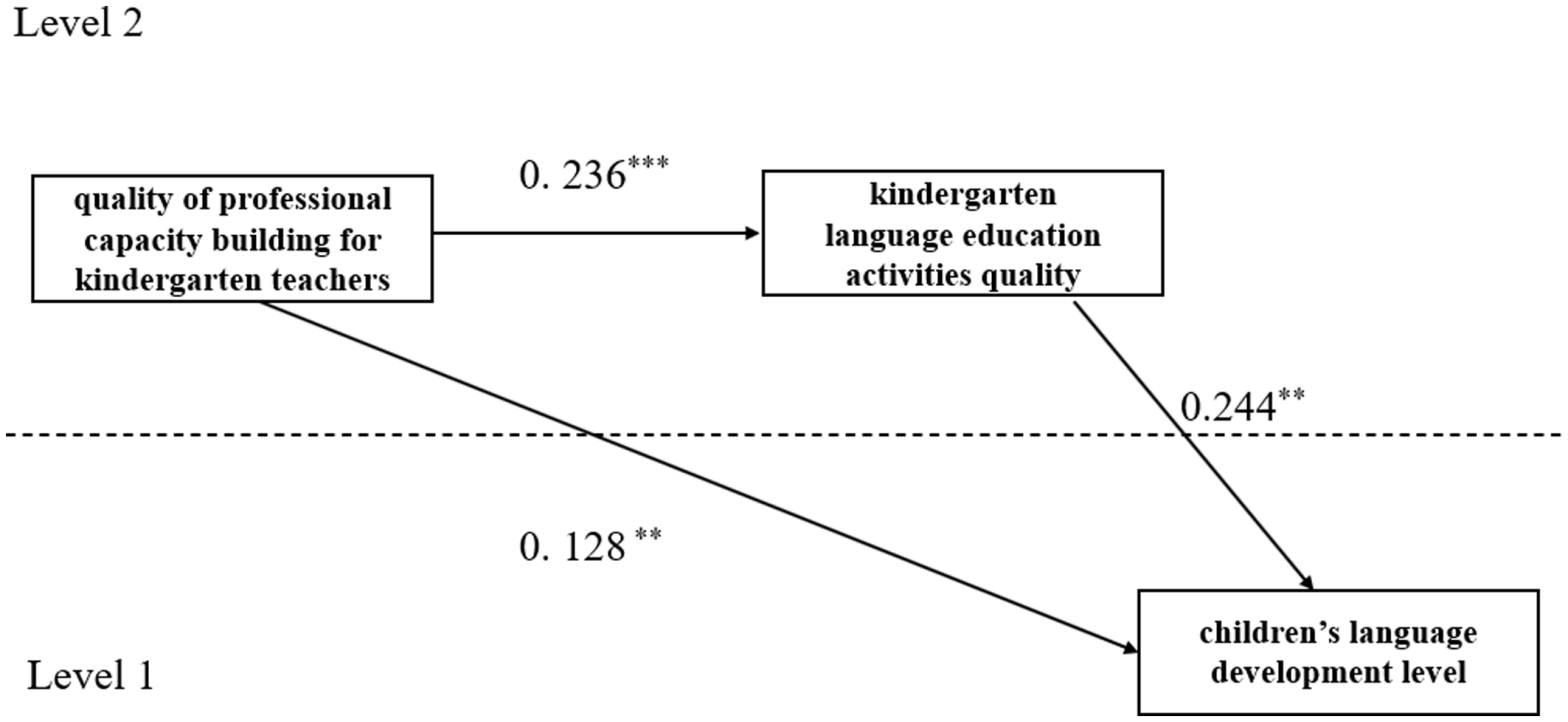
Mediating effect analysis of the kindergarten language education activities quality between quality of professional capacity building for kindergarten teachers and children’s language development level.

The following are the steps for analyzing the mediating effect of kindergarten language education activities quality between the quality of professional capacity building for kindergarten teachers and children’s language development level, using a multilevel structural equation modeling approach:

Step 1: Multilevel mediation model (see Figure 1) is tested, with quality of professional capacity building for kindergarten teachers as the independent variable, kindergarten language education activities quality as the mediator, and children’s language development level as the dependent variable. The model fit indices are as follows: CFI = 1.00, TLI = 1.00, RMSEA<0.001, SRMRB = 0.000, SRMRw<0.001, indicating good model fit.

Step 2: The results of the multilevel structural equation modeling analysis indicate that the path coefficient from quality of professional capacity building for kindergarten teachers to the kindergarten language education activities quality (a) is significant (*γ* = 0.236, *p*<0.001). The path coefficient from the kindergarten language education activities quality to children’ s language development level (b) is significant (*γ* = 0.244, *p*<0.01). The mediating effect of *a* × *b* is significant (*γ* = 0.058, *p*<0.001), and the direct effect from the quality of professional capacity building for kindergarten teachers to children’ s language development level (c) is also significant (*γ* = 0.128, *p*<0.01) (see Table 4). This indicates that kindergarten of language education activities quality partially mediates the relationship between quality of professional capacity building for kindergarten teachers and children’s language development level in a 2-2-1 model.

**Table 4:** Hypotheses model path coefficient.

Model pathway	Point estimate	95% CI	S.E.	Est./S.E.	*p*
Quality of professional capacity building for kindergarten teachers➔kindergarten language education activities quality	0.236	[0.139, 0.334]	0.059	3.979	<0.001
Kindergarten language education activities quality➔children’s language development level	0.244	[0.121, 0.366]	0.074	3.282	<0.01
Quality of professional capacity building for kindergarten teachers➔children’s language development level	0.128	[0.061,0.195]	0.041	3.129	<0.01
Quality of professional capacity building for kindergarten teachers➔kindergarten language education activities quality➔kindergarten language education activities quality	0.058	[0.031, 0.084]	0.016	3.546	<0.001

## 4. Discussion

### 4.1. Significant differences in language development levels exist among children in kindergartens of different characteristics

This study reveals that significant differences exist in language development levels among children attending kindergartens of different characteristics in China. Children in public kindergartens exhibit considerably higher language development levels than those in private kindergartens. This is consistent with previous research findings. For instance, studies have shown that children in public kindergartens score significantly higher in areas such as free play, language, and social domains compared to those in private kindergartens (Liu et al., 2008). Other research indicates that children in private kindergartens score lower in language, mathematics, science, and social domains than their public kindergarten counterparts (Shi and Ye, 2016).

Various factors contribute to these differences in children’s language development levels, with disparities in educational quality being a crucial factor. Studies have shown that high-quality kindergarten education can bring positive impacts on children’s future development (Nores et al., 2005). The higher the quality of kindergarten education, the greater the added value for children’s development (Vandell et al., 2010); Therefore, China should strive to improve the educational quality of private kindergartens. On one hand, the proportion of funding for inclusive private kindergartens should be clarified, providing adequate financial support for the supply of high-quality inclusive educational resources. On the other hand, the quality supervision and dynamic regulation of private kindergartens should be strengthened, with periodic diagnostic assessments and rectifications, linking quality assessment results with funding subsidies to motivate private kindergartens to continuously enhance their educational quality.

### 4.2. The quality of professional capacity building for kindergarten teachers is significantly positively correlated with children’s language development levels

This study finds that quality of professional capacity building for kindergarten teachers is significantly positively correlated with children’s language development levels. These findings are consistent with previous research perspectives. Some studies have pointed out that research support significantly affects the added value of children’s development in language and early reading domains, especially for children with lower initial levels in language and early reading (Li et al., 2021). Simultaneously, the results of this study also validate the theoretical perspectives of some researchers. Some researchers believe that research activities can enhance the quality of kindergarten educational activities and improve children’s development outcomes (Huang, 2019); kindergarten-based research is an essential approach to promoting kindergarten teachers’ professional development, serving as an essential platform for their professional growth and a mediator for translating educational concepts into educational practices, playing a crucial role in improving kindergarten education quality and promoting children’s development (Zou and Hua, 2015). Autonomous learning of kindergarten teachers not only enhances their professional literacy and kindergarten education quality but also fosters the comprehensive and healthy development of children (Hai and Liu, 2018). Therefore, kindergartens should solidly carry out teacher training, effectively organize teaching and research activities, implement teacher incentives and evaluation, and promote teacher autonomous development. Through the effective implementation of these activities, the continuous improvement of teachers’ professional capacity can be promoted, thereby providing robust support for children’s language development.

### 4.3. The kindergarten language education activities quality plays a partial mediating role between the quality of professional capacity building for kindergarten teachers and children’s language development levels

This study demonstrates that the kindergarten language education activities quality plays a partial mediating role between quality of professional capacity building for kindergarten teachers and children’s language development levels. In other words, the quality of professional capacity building for kindergarten teachers not only directly affects children’s language development levels but also indirectly influences them through the impact on the kindergarten language education activities quality. This suggests that teachers can apply the teaching experiences or strategies gained from professional capacity building activities to the implementation of kindergarten language education activities, thereby promoting children’s language development. This validates existing research perspectives, as studies have indicated that teachers’ sensitivity and effective responses to children’s behavior during the organization and implementation of educational activities can effectively predict children’s language and social interaction abilities (Burchinal et al., 2008). Based on this, kindergartens can focus on the implementation of language education activities, evaluate teachers’ performance in designing and implementing language education activities, examine the quality of professional capacity building for kindergarten teachers, and encourage teachers to translate their acquired knowledge and skills from understanding and acceptance to practical implementation, ultimately promoting children’s language development.

## 5. Conclusion

Firstly, the significant differences exist in the language development levels of children attending kindergartens with different types. Children in public kindergartens demonstrate notably higher language development levels compared to those in private kindergartens.

Secondly, the quality of professional capacity building for kindergarten teachers has a significant positive predictive effect on children’s language development levels. Improving the quality of professional capacity building for kindergarten teachers can enhance children’s language development levels.

Thirdly, kindergarten language education activities quality plays a partial mediating role between the quality of professional capacity building for kindergarten teachers and children’s language development levels. That is, the quality of professional capacity building for kindergarten teachers affects children’s language development levels through two pathways: directly influencing children’s language development levels and indirectly affecting children’s language development levels by influencing kindergarten language education activities quality.

## 6. Limitations of the study and future research directions

Several limitations of the present study warrant note. First, our evaluation tool for quality of professional capacity building for kindergarten teachers may not have been sensitive enough to reflect the overall performance of kindergarten teachers’ professional capacity building. Nevertheless, we believe that the evaluation tool supports the validity of our findings. Because the tool covers many key aspects of professional capacity building for kindergarten teachers, the score was obtained on the basis of on-site observation and data review.

Secondly, the study only focuses on the relationship between kindergarten language education activities quality, the quality of professional capacity building for kindergarten teachers and children’s language development. There may be other situations that lead to children’s language development, such as other areas education activities in kindergarten, more and higher quality dialogue between teachers and children, which need to be further studied.

Thirdly, this study only made a brief analysis of language development levels exist among children in public kindergartens and private kindergartens. Future studies should deeply explore the factors affecting children’s language development levels in private kindergartens and take effective measures to support these children.

## Data availability statement

The raw data supporting the conclusions of this article will be made available by the authors, without undue reservation.

## Ethics statement

The studies involving human participants were reviewed and approved by Institutional Review Board of Northeast Normal University. Written informed consent to participate in this study was provided by the participants’ legal guardian/next of kin.

## Author contributions

QW and GW designed the study and devised the project. QW and CL took the lead in writing the manuscript. GW verified the analytical methods and contributed to manuscript revision. All authors contributed to the article and approved the submitted version.

## Funding

This study was supported by Humanities and Social Science of Ministry of Education Planning Fund: “Research on Strategies for Improving the Quality of Teacher Child Interaction in Kindergartens” (Reference No.: 19YJA880065). This study was also partially funded by China National Society of Early Childhood Education key topics in the 13th Five-Year Plan: “Research on the Evaluation Standards of China’s High Quality Kindergartens.”

## Conflict of interest

The authors declare that the research was conducted in the absence of any commercial or financial relationships that could be construed as a potential conflict of interest.

## Publisher’s note

All claims expressed in this article are solely those of the authors and do not necessarily represent those of their affiliated organizations, or those of the publisher, the editors and the reviewers. Any product that may be evaluated in this article, or claim that may be made by its manufacturer, is not guaranteed or endorsed by the publisher.
